# Similar fish species composition despite larger environmental heterogeneity during severe hypoxia in a coastal ecosystem

**DOI:** 10.1002/ece3.8884

**Published:** 2022-05-15

**Authors:** Naoto Shinohara, Yuki Hongo, Momoko Ichinokawa, Shota Nishijima, Shuhei Sawayama, Hiroaki Kurogi, Yasuyuki Uto, Hisanori Mita, Mitsuhiro Ishii, Akane Kusano, Seiji Akimoto

**Affiliations:** ^1^ 13519 Fisheries Resources Institute Japan Fisheries Research and Education Agency Yokohama Japan; ^2^ 97556 Marine Industries Promotion Division Chiba Prefectural Government Chiba Japan; ^3^ 53333 Chiba Prefectural Fisheries Research Center Chiba Japan; ^4^ 97553 Fisheries Division Environment and Agriculture Bureau Kanagawa Prefectural Government Yokohama Japan; ^5^ Fisheries Research Institute of Kanagawa Prefecture Yokohama Japan

**Keywords:** community assembly, eDNA, environmental filtering, environmental heterogeneity, fish, hypoxia

## Abstract

Environmental heterogeneity is one of the most influential factors that create compositional variation among local communities. Greater compositional variation is expected when an environmental gradient encompasses the most severe conditions where species sorting is more likely to operate. However, evidence for stronger species sorting at severer environment has typically been obtained for less mobile organisms and tests are scarce for those with higher dispersal ability that allows individuals to sensitively respond to environmental stress. Here, with the dynamics of fish communities in a Japanese bay revealed by environmental DNA metabarcoding analyses as a model case, we tested the hypothesis that larger environmental heterogeneity caused by severe seasonal hypoxia (lower concentration of oxygen in bottom waters in summer) leads to larger variation of species composition among communities. During summer, fish species richness was lower in the bottom layer, suggesting the severity of the hypoxic bottom water. In contrast to the prediction, we found that although the environmental parameters of bottom and surface water was clearly distinct in summer, fish species composition was more similar between the two layers. Our null model analysis suggested that the higher compositional similarity during hypoxia season was not a result of the sampling effect reflecting differences in the alpha or gamma diversity. Furthermore, a shift in the species occurrence from bottom to surface layers was observed during hypoxia season, which was consistent across species, suggesting that the severe condition in the bottom adversely affected fish species irrespective of their identity. These results suggest that larger environmental heterogeneity does not necessarily lead to higher compositional variation once the environmental gradient encompasses extremely severe conditions. This is most likely because individual organisms actively avoided the severity quasi‐neutrally, which induced mass effect‐like dispersal and lead to the mixing of species composition across habitats. By showing counter evidence against the prevailing view, we provide novel insights into how species sorting by environment acts in heterogeneous and severe conditions.

## Graphical abstract

Species compositional variation is generally believed to be more dissimilar when the environment is heterogeneous. Against this prediction, we found that the coastal fish species composition was less diverged during hypoxia season when the environment was more heterogeneous. This was likely because the severest condition lead to more neutrality or induced mass effect‐like dispersal.

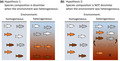

## 1 INTRODUCTION

Understanding the processes controlling the variation in species composition across multiple habitats has been a central challenge in ecology. The metacommunity framework (Leibold et al., 2004; Thompson et al., 2020) provides a way to integrate multiple community assembly processes and roles played by environmental heterogeneity and dispersal. The species sorting paradigm, one of the paradigms in the metacommunity framework, predicts that species composition deterministically aligns with environmental gradients as determined by each species’ requirements for some abiotic conditions (Grinnell, 1917; Tilman, 1982). In this paradigm, species composition is expected to be more dissimilar if environmental heterogeneity among habitats is larger (MacArthur et al., 1966; Ricklefs, 1977). Meanwhile, the mass effect paradigm considers the higher rate of dispersal of individuals, which promotes the mixing of species composition among localities (Mouquet & Loreau, 2002). In this case, similar species composition may be observed even among environmentally heterogeneous habitats. The neutral dynamics paradigm, where species are modeled as equivalent in their fitness and competitive ability, poses a prediction similar to the mass effect that the environment–species composition relationship is no longer detectable. These paradigms are not discretely separated; rather, they are considered as the continuum determined by the dispersal rate and the degree of interspecific differences (Thompson et al., 2020). In this context, much effort has been devoted to understanding the condition in which the relative importance of species sorting by environment over the mass effect or neutrality varies (Chase & Myers, 2011; Gravel et al., 2006).

**FIGURE 1:**
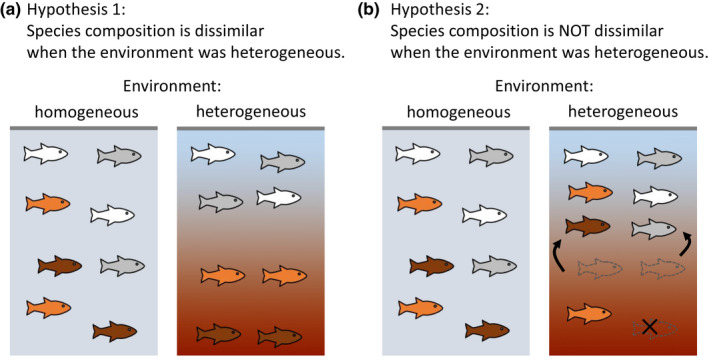
Two alternative hypotheses regarding the effect of environmental heterogeneity on variation in species composition. (a) Habitats with distinct environments (e.g., different water layers) have more dissimilar species composition when the environmental heterogeneity is large because of species sorting. (b) Even habitats with distinct environment share similar species composition because the severest conditions (e.g., extreme hypoxia at the bottom) induce higher mortality rates or movement toward preferable habitats (e.g., the surface layer) irrespective of species identity

Growing body of literature suggests that species sorting plays a more important role when the abiotic environment is more severe (Chase, 2007; Daniel et al., 2019; Guo et al., 2014; Lepori & Malmqvist, 2009). This is because at the severe environment, where the growth rate of species is lowered or the mortality rate is high, only a limited number of species that possess traits adapted to the severe environment can survive. This leads to a prediction that compositional variation becomes great when environmental heterogeneity is large enough to encompass the severe environment where species sorting is more likely to act (Figure 1a). However, there are two major knowledge gaps in the previous literature, which hinders our ability to fully understand the relationship between environmental severity and community assembly.

First, the current understanding assumes that responses to environmental severity differ among species. However, a limited number of studies indicated exceptional cases in which species difference becomes small and species sorting is weak at the severest conditions (Kim, 2019; Lepori & Malmqvist, 2009). For example, Lepori and Malmqvist (2009) found that although moderate disturbance in streams (frequent flooding) filtered out some species that are intolerant of the imposed stress, too severe disturbance did not work as species sorting because it induced local extinction of individuals irrespective of species traits. Therefore, the effect of environmental severity on the strength of species sorting depends on whether the species difference is maintained at the most stressful conditions, but little attention has been paid for this possibility in the current framework (Lepori & Malmqvist, 2009). The second knowledge gap is that the evidence that species sorting is more likely at the severe environment typically comes from systems where dispersal of individuals is limited or passive, such as plants or aquatic organisms in ponds (e.g., Chase, 2007; Guo et al., 2014), and little is known for organisms with higher dispersal ability. Unlike less mobile organisms, highly mobile organisms can actively escape from unpreferable severe environments, which can lead to the concentration of many species in preferable habitats, regardless of their ability to tolerate the stress. In that case, while the environmental severity indeed affects organisms’ distribution, it induces the mass effect‐like dispersal and no longer operates as ‘species’ sorting. Therefore, when the environmental heterogeneity becomes large enough to encompass the severest conditions, it is probable that the compositional variation would not be larger or even be smaller despite the environmental heterogeneity (Figure 1b), due to smaller species differences (i.e., more neutrality) or nonspecies‐specific movement toward preferable habitats (i.e., mass effect‐like dispersal).

In marine ecosystems, one of the environmental conditions that most drastically influence community structures is hypoxia or the decline in dissolved oxygen (DO; Breitburg et al., 2018; Diaz & Rosenberg, 1995, 2008). One of the typical forms of hypoxia is seasonal: in summer, water warming forms the stratification of water columns, which leads to the environmental heterogeneity along water depth. Specifically, the reduced concentration of DO in the bottom water to a critical level imposes extreme stress for demersal organisms, such as increased mortality rates (Hrycik et al., 2017; Levin, 2003). Therefore, the species‐sorting paradigm (Figure 1a) expects that species that do not possess morphological or physiological traits adapted to the hypoxic conditions are likely to be filtered out from the bottom water (Bickler & Buck, 2007; Kodama & Horiguchi, 2011; Levin, 2003), and different species composition between bottom and surface water is expected during that season. In contrast, it is also probable that the bottom hypoxia was so intense that many species suffer from it or actively avoid it to a similar extent (Eby & Crowder, 2002; Pihl et al., 1991), and species can be apparently neutral with respect to the responses to the severe hypoxia, weakening the effect of species sorting (Figure 1b).

The objective of this study is to explore whether among‐habitats compositional variation becomes larger when environmental heterogeneity is large enough to encompass the most severe condition. We focused on the influence of bottom hypoxia in summer on fish communities in Tokyo Bay, one of the largest bay areas in Japan, as a model case. The water stratification in summer causes hypoxic water mass at bottom with a size in height of ~30 m over a wide area within the bay (Ishii & Ohata, 2010; Yasui et al., 2016) which severely affects organisms in the bottom waters (Kodama & Horiguchi, 2011). We have two alternative hypotheses: (1) species composition is more dissimilar between bottom and surface layers during the hypoxia season (summer), because the environmental heterogeneity is larger, and the bottom environment is extremely stressful which strongly sorts species that are adapted to the severity (Figure 1a). Alternatively, (2) compositional difference between bottom and surface waters is not evident or even smaller during hypoxia season despite larger environmental heterogeneity. This is because the extremely severe environment in the bottom negatively affects fish occurrences irrespective of the species identity and thus the severity does not operate as ‘species’ sorting (Figure 1b), which leads to the mixing of species composition. To this end, we took advantage of the availability of data of seasonal dynamics of fish communities in Tokyo Bay revealed by the environmental DNA (eDNA) metabarcoding analysis (Hongo et al., 2021) which has emerged as a powerful and efficient tool for monitoring biodiversity especially in aquatic ecosystems (Bohmann et al., 2014; Deiner et al., 2017). By extracting genetic materials shed from organisms from environmental samples (e.g., water and soils), this technique allows for the noninvasive assessment of biodiversity, with a detection ability comparable or superior to that of traditional sampling methods (Sigsgaard et al., 2017; Thomsen et al., 2012; Yamamoto et al., 2017). Importantly, owing to the short persistence (from a few hours [Murakami et al., 2019] to several days [Thomsen et al., 2012]) and low diffusion rate (less than 100 m; Port et al., 2016; Murakami et al., 2019) of eDNA in seawater, it enables the estimation of contemporary and local species composition (Yamamoto et al., 2016). This technique allowed for frequent sampling of fish communities throughout a single year in both bottom and surface waters having different environments to test the alternative hypotheses on the effect of environmental severity on local species composition in a metacommunity (Figure 1).

## 2 MATERIALS AND METHODS

### 2.1 Water sampling and eDNA sequencing

Water sampling was conducted monthly at 14 sites across Tokyo Bay, Japan (approximately 1380 km^2^, 35°30′N, 139°50′E, Figure S1a) from January to December 2019. At each sampling location, 1 L of water was collected from the surface and bottom layers. Bottom depth varied among sites from approximately 6 to 70 m (Figure S1a). Water samples were filtered and frozen onboard a ship. At end of sampling, 1 L of distilled water was filtered as a negative control and stored with the frozen filter samples. The frozen filters were then transferred to and stored in the laboratory. The extraction of eDNA and the construction of amplicon sequencing variants (ASVs) were performed as described by Hongo et al. (2021) using MiFish universal primers (Miya et al., 2015). One sample replicate on the field, and one PCR replicate per sample was conducted in this study. The constructed ASVs were assigned to fish species using Blastn against the MitoFish database (Sato et al., 2018). As the correlation between eDNA concentration and abundance of target fish species is variable under natural conditions (Yates et al., 2019), the count data of ASVs was replaced to presence/absence data. The eDNA sequence data are available at the DNA Data Bank of Japan Sequence Read Archive under the accession number DRA010909.

### 2.2 Environmental variables

**FIGURE 2:**
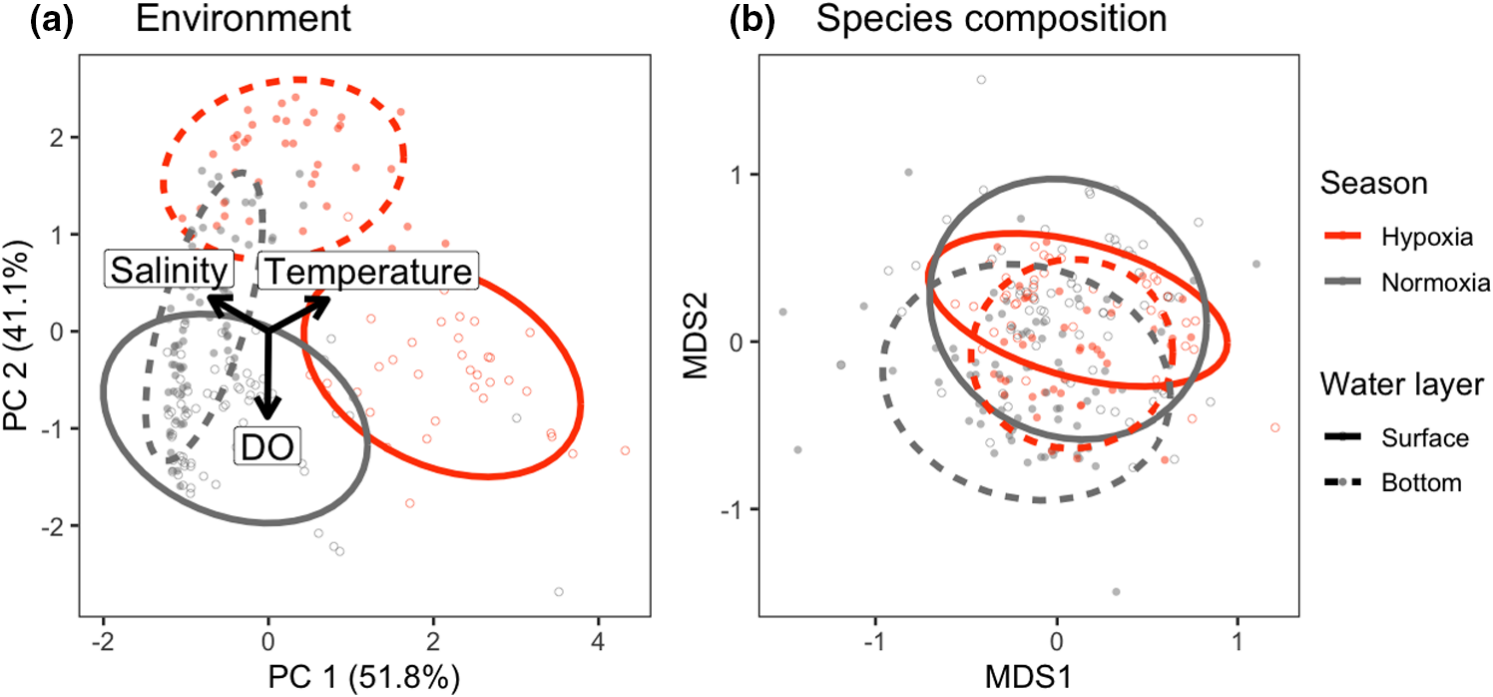
(a) Result of the PCA on three environmental variables (DO concentration, water temperature, and salinity). Arrows represent the contribution of the variables on the PC space. Each ellipse represents the 80% confidence level of the samples of each category (layer and season) in the first two PC axes, with its color and line type corresponding to season (orange: hypoxia, black: normoxia) and layer (solid: surface, broken bottom), respectively. Background points similarly depict the samples, with the color and point type corresponding to season and layer (open: surface, filled: bottom), respectively. (b) Result of NMDS on species composition based on Jaccard dissimilarity (dimension = 3, stress = 0.190). The ellipses and points are drawn in the same way as in (a)

Three environmental variables, namely concentration of DO, water temperature, and salinity, were measured at the same time as the water sampling. During summer, the water temperature and DO concentration were lower in the bottom than in the surface (Figures 2a and S1b). Specifically, from June to September, the minimum value of DO concentration was lower than 2 mg/L (Figure S1b), a common threshold of hypoxia in previous studies (Breitburg et al., 2018; Hrycik et al., 2017; Pihl et al., 1991). Following the established threshold in the literature, we determined these months as “hypoxia” season and other months as “normoxia” (normal levels of oxygen). Note that even in the hypoxia season, the DO values considerably varied among samples, with many being higher than the threshold (Figure S1b). Nevertheless, we classified all samples of the months when the minimum DO value falls below the threshold as the hypoxia season. This is because analyzing the samples obtained in the same period as one unit allows us to evaluate the effects of environmental heterogeneity on the compositional variation among samples.

In addition, while significant decreases in DO concentration in summer were observed at most of the sites, this was not the case at sites near the mouth of the bay (Figure S1) because of deep water depth and water exchange with the ocean (Fujiwara & Yamada, 2002), which may unexpectedly blur the results. Nevertheless, our main finding was unchanged when we excluded the sampling sites near the bay mouth from the analyses (Figure S2).

### 2.3 Data analysis

To minimize unexpected biases, we filtered out the species that did not meet the following two criteria from further analyses: (1) those whose names were not matched during the search in Fishbase, a global database of fish (Froese & Pauly, 2019; https://www.fishbase.se/), and (2) those whose habitats were known to be out of Tokyo Bay based on information from a range of studies (Kouno et al., 2011; Nakabo, 2002). The first criterion mainly filtered out hybrid species. The second one, which was based on each fish species’ distribution in Japan and observation records in Tokyo Bay, removed fish species known to be freshwater or endemic in other regions which were detected likely due to sequencing errors or contamination from rivers or nearby fish markets.

All statistical analyses were performed using R version 4.0.2 (R Core Team, 2020) and the figures were illustrated using the *ggplot2* package (Wickham, 2016). First, to examine whether the environment at the bottom during hypoxia was severe for fish species, we constructed generalized linear mixed models (GLMMs) with Poisson distribution (log link) that modeled the fish species richness as a function of season (categorical: hypoxia or normoxia), water layer (categorical: surface or bottom), and their interactive effect. Identity of sampling sites was incorporated as a random effect. We expected that if the environment at the bottom water was severe during hypoxia, species richness would be lowered by the interactive effect (hypoxia × bottom). The models were implemented using the *glmmTMB* function in the *glmmTMB* package (Brooks et al., 2017).

Second, to examine the environmental and compositional heterogeneity between bottom and surface layers, we conducted a principal components analysis (PCA) on the three environmental variables (DO concentration, water temperature, and salinity) and nonmetric multidimensional scaling (NMDS) on the species composition data (both *n* = 226). These allow the environmental and compositional dissimilarity among communities to be interpreted in a low‐dimensional space by positioning similar data nearby. For the NMDS analysis, we used the Jaccard dissimilarity index on the presence–absence dataset and three dimensions (*k* = 3) to facilitate convergence. For the visualization, we plotted values of the first two PC axes of the environmental variables, which explained 51.8% and 41.1% of the total variation respectively, and the first two MDS axes of the species composition. Differences in the environment and composition among seasons and layers were tested using permutational multivariate analysis of variance (PERMANOVA; Anderson, 2001), based on Euclidean and Jaccard distance metrices for the environment and composition data, respectively. We included the interaction term between season and layer, as well as their independent effects, in the PERMANOVAs to determine whether there exists a seasonal difference in the extent of surface‐bottom divergence. Since the number of sampling sites was small (*n* = 2) due to logistical problems, we did not conduct the above analyses for the October data. The PERMANOVAs were implemented by the *adonis* function in the *vegan* package (Oksanen et al., 2019).

Results of the PERMANOVA that tested the interactive effect of season and layer on species composition may be biased by seasonal differences in the size of regional species pool (gamma‐diversity) and local species richness (alpha‐diversity), because higher gamma‐diversity and lower alpha‐diversity generally lead to larger compositional difference simply due to the sampling effect (Kraft et al., 2011; Mori et al., 2015). To examine this undesired possibility, we relied on a null model analysis. Our null model randomly shuffled the occurrence of species in each sample with total number of occurrences of each species and the samples’ species richness fixed. Note that the randomization was conducted separately for each month as a unit sharing the same regional species pool. For each month, 999 randomized community matrices (sample × species) were generated based on the matrix‐swap algorithm (Gotelli, 2000). The randomization was implemented by the *RandomizeMatrix* in the package *picante* (Kembel et al., 2010). For each pair of samples, Jaccard dissimilarity index was calculated for the 999 randomized matrix to obtain its null distribution. From the null distribution, we evaluated where the observed dissimilarity index lies in the quantiles (%) of the null distribution. A larger quantile value suggests that the pair of samples is compositionally more dissimilar after accounting for the sampling effect. We compared the quantile values of sample pairs in hypoxia and normoxia seasons using Wilcoxon rank sum test. We expect that if compositional variation was larger during hypoxia even after accounting for the sampling effect, as predicted from our first alternative hypothesis, then more pairs would be more dissimilar and thus the quantile values be higher during hypoxia. Our second hypothesis predicts that the degree of compositional variation is similar between hypoxia and normoxia or smaller during hypoxia and so the quantile values are comparable between seasons or lower during hypoxia.

To examine the species‐specific responses to bottom hypoxia, we compared the frequency of occurrence of fish species between seasons (hypoxia or normoxia) and water layers (surface or bottom), using a GLMM with species‐specific random intercept and coefficients. The response variable was the presence probability of species *i* in a sample *s* (*p_i_*_,_ *_s_*), and the explanatory variables were season, layer and their interaction:

$$\text{logit}\;\left(p_{i,s}\right)=\beta_{0,i}+\beta_{1,i}\times {\text{Season}}_{s}+\beta_{2,i}\times {\text{Layer}}_{s}+\beta_{3,i}\times {\text{Season}}_{s}\times {\text{Layer}}_{s}.$$

The season and layer were transformed into dummy variables (hypoxia: 0, normoxia: 1, and surface: 0, bottom: 1). To assure sufficient statistical power, we restricted this analysis to the frequently observed species, that is, top 10% most frequently observed species (17 out of 168 species). Therefore, the dataset used in this GLMM consisted of 3842 presence or absence records (17 species × 226 samples). The species‐specific intercept (*β*_0,_ *_i_*) and coefficients (*β*_1,_ *_i_*, *β*_2,_ *_i_*, and *β*_3,_ *_i_*) were modeled to follow normal distributions, reflecting that the baseline of occurrence probability, effects of season, water layer and their interaction on the probability may differ among species. In light of our hypothesis, the significantly positive coefficient of the fixed interaction term (*β*_3,_ *_i_*) implies that the relative occurrence in the bottom over surface layer was higher in normoxia on average across species. To further test the hypothesis on species‐specific responses to bottom hypoxia, we additionally constructed a GLMM similar to the previous one except for that the coefficient of the interaction term (*β*_3,_ *_i_*) was modeled as a fixed effect (*β*_3_). We compared the fittings of the two GLMMs by the likelihood ratio test using the *anova* function, which informs us whether modeling the species‐specific responses to bottom hypoxia improves the model. If the test indicated no significant differences, we conclude that the effect of season‐depth interaction can be modeled as common across species.

Finally, we examined whether the compositional data obtained by eDNA metabarcoding analyses was influenced by the vertical mixing of eDNA. While the eDNA signal is known to provide information regarding vertical distribution of species, its spatial resolution is unclear in bay systems. As our sample sites vary in their water depth (approximately from 6 to 70 m), vertical mixing of eDNA potentially biases the detected compositional variation between bottom and surface waters at shallower sites. To examine this potential bias, we conducted PERMANOVA based on Jaccard dissimilarity index on species composition data with the water layer (categorical: surface or bottom) and the depth of the sampling site (continuous) and their interactive term as explanatory variables. If vertical mixing occurs, we expect the compositional difference between bottom and surface layers is smaller at shallower sites and thus the interactive term is significant.

## 3 RESULTS

We obtained 308 water samples for the eDNA extraction, and gene amplification were successful for 281 samples, from which about 28 M reads were obtained and approximately 4 M reads were assigned as fish species (Hongo et al., 2021). The gene amplifications were not detected from negative control filters. Among these, corresponding environmental data were available for 226 samples (*n* = 23, 22, 22, 21, 20, 20, 18, 21, 20, 2, 15, and 22 from January to December, respectively). A total of 220 fish species were detected in the 226 samples. Of these, our first criterion filtered out 12 species, and the second criterion eliminated 40 species, resulting in 168 species retained in the subsequent analyses (Table S1).

During normoxia, species richness per sample was higher in the bottom layer (mean: 10.1, standard deviation [SD]: 4.78, Figure S3) than in the surface layer (mean: 6.24, SD: 3.05). However, such a difference was small during hypoxia: mean species richness was 9.15 (SD: 4.78) in the bottom and 8.13 (SD: 3.16) in the surface. Such an interactive effect of season and water layer was significant in the GLMM (hypoxia:bottom, coefficient = −0.351; *p* < .001), as well as independent effects of water layer (bottom, coefficient = 0.474; *p* < .001) and season (hypoxia, coefficient = 0.258, *p* < .001).

The environment was significantly different between seasons (hypoxia vs. normoxia, *F*_1, 225_ = 125.8; *R* ^2^ = 0.211; *p* < .001, PERMANOVA, Figure 2a) and between water layers (surface vs. bottom, *F*_1, 225_ = 212.4; *R* ^2^ = 0.356; *p* < .001). In addition, there was a significant interactive effect of season and water layer (*F*_1, 225_ = 35.8; *R* ^2^ = 0.060; *p* < .001), suggesting that the bottom‐surface environmental heterogeneity was larger during hypoxia.

The species composition significantly differed between layers (surface vs. bottom, *F*_1, 225_ = 9.21; *R* ^2^ = 0.039; *p* < .001, PERMANOVA, Figure 2b), and seasons (hypoxia vs. normoxia, *F*_1, 225_ = 5.37; *R* ^2^ = 0.022; *p* < .001). Furthermore, their interactive effect was also significant (*F*_1, 225_ = 2.58; *R* ^2^ = 0.011; *p* < .001), suggesting that the degree of surface‐bottom divergence differed between the two seasons. The compositional difference between surface and bottom layers was smaller in hypoxia, as the distance in the three‐MDS dimensions between the centroids of the surface and bottom samples was 0.27 for hypoxia, and 0.50 for normoxia (Figure 2b).

**FIGURE 3:**
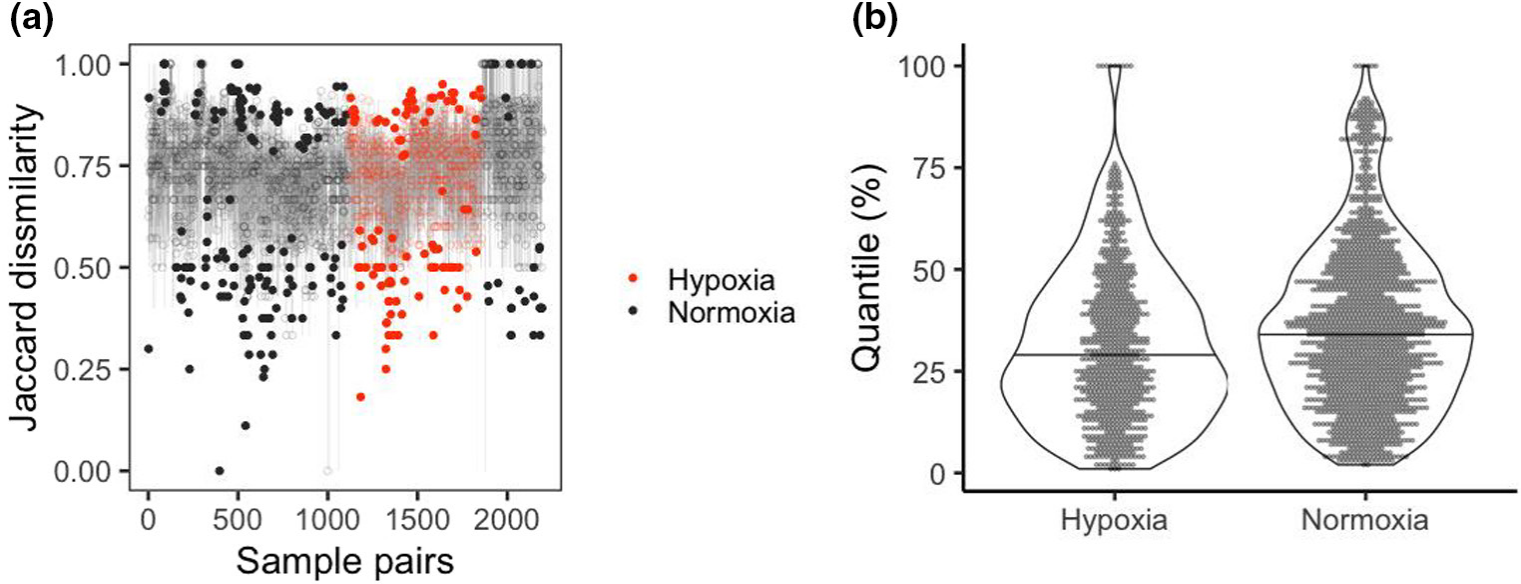
Results of our null model analysis on the Jaccard dissimilarity index. (a) The observed values (points) and null distribution (gray vertical lines, between 10% and 90% quantiles of the null values) of the index of sample pairs. The dissimilarity index was calculated for all the pairs of the same months (2194 pairs in total). Sample pairs are ordered in the *x*‐axis according to the sampling month from left (January) to right (December). Pairs of samples in the hypoxia and normoxia season are colored with orange and black, respectively. Points are filled when the observed value is larger than the 90% quantile or smaller than the 10% quantile. (b) Between‐seasons comparison of the quantile values of the observed index in its null distribution. The average of quantile values is smaller during hypoxia (*p* < .001, Wilcoxon rank sum test)

Observed compositional dissimilarity (Jaccard index) of most of the sample pairs were within the range between the 10% and 90% quantiles of their null distribution (638 out of 743 pairs in hypoxia, and 1266 out of 1451 pairs in normoxia, Figure 3a), suggesting that the observed dissimilarity indices were not strongly deviated from the null expectation. Nevertheless, the quantile values of the observed index in its null distribution were smaller during hypoxia (*p* < .001, Wilcoxon rank sum test, Figure 3b), supporting the above result that compositional dissimilarity is smaller during hypoxia (Figure 2b).

**FIGURE 4:**
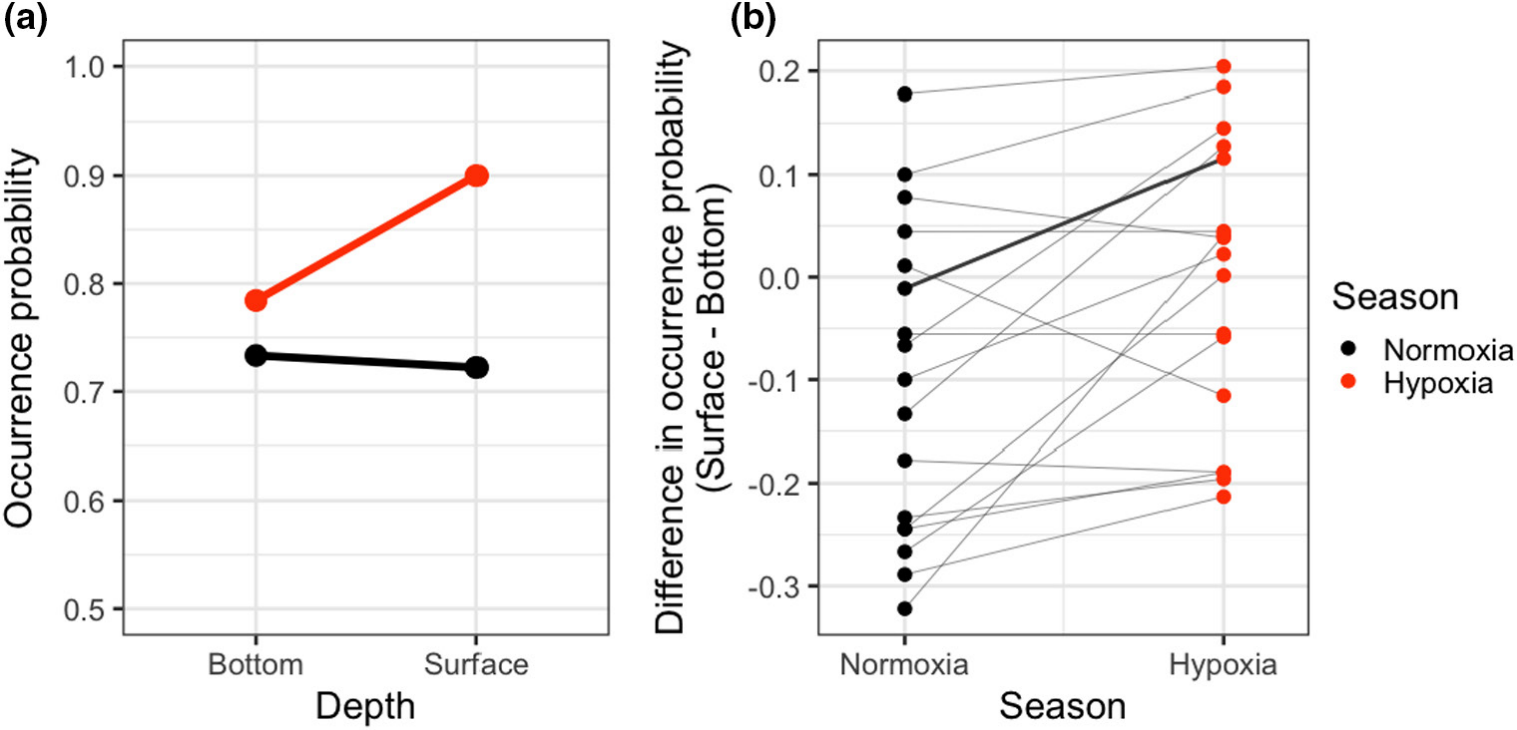
Observed relative occurrence probability in the surface and bottom layers of fish species in hypoxia and normoxia. (a) An example for *Konosirus punctatus*, the second most frequently observed species. The relative occurrence in the surface compared to the bottom layer is higher during hypoxia. (b) Differences in the occurrence probability in the surface and bottom layers in different seasons of the top 10% most frequently observed species (17 species). The points representing the same species are linked with lines, and a bold line corresponds to the example in (a). The increase in the relative occurrence in the surface layer during hypoxia was observed for 14 species

Relative occurrence frequency of fish species in the surface and bottom layers differed between hypoxia and normoxia. For example, *Konosirus punctatus*, the second most frequently observed species (216 out of 281 samples), occurred in the bottom water (73.3%, Figure 4a) slightly more frequently than in the surface (72.2%) in normoxia. However, this tendency was reversed in hypoxia, and it more frequently occurred in the surface (90.0%) than in the bottom (78.4%). Among the top 10% most frequently observed species (*Engraulis japonicus*, *Konosirus punctatus*, *Lateolabrax japonicus*, *Hemitrygon akajei*, *Mugil cephalus*, *Acanthopagrus schlegelii*, *Pennahia argentata*, *Callionymus valenciennei*, *Sardinella lemuru*, *Scomber japonicus*, *Sardinops sagax*, *Scomberomorus niphonius*, *Seriola quinqueradiata*, *Trachurus japonicus*, *Mustelus manazo*, *Branchiostegus japonicus*, and *Trichiurus lepturus*, in order of occurrence frequency), such an interactive effect of depth and season on occurrence (i.e., an increase of the relative occurrence in the surface in hypoxia) was observed for 14 out of 17 species, excepting for Japanese anchovy (*Engraulis japonicus*), Japanese sea bass (*Lateolabrax japonicus*), and Chub mackerel (*Scomber japonicus*) (Figures 4b and S4). The GLMM, where the occurrence of each fish species was modeled as a function of water layer, season, and their interaction with species‐specific random intercept and slopes, showed that layer and season did not influence the occurrence probability averaged across the species (layer: coefficient = 0.228; standard error [SE] = 0.225; *p* = .311, season: coefficient = −0.409; SE = 0.216, *p* = .058, Table 1a). Nevertheless, their fixed interactive term was significant (coefficient = 0.643; SE = 0.228; *p* = .005) with smaller random variance (0.328) than coefficients of the independent effects (layer: 0.489, season: 0.462). Indeed, the estimated species‐specific coefficients of the interaction term were positive for all the 17 species (Figure S5). Furthermore, the likelihood test that compared the above GLMM with another model without the species‐specific random effect for the interaction term (Table 1b), showed no significant differences between the models (*χ* ^2^ = 2.56, df = 1, *p* = .109). These results collectively imply that the negative effect of hypoxia at bottom on the occurrence probability can be regarded as consistent across species.

**TABLE 1:** Results of the two GLMMs that modeled the presence of species as a function of water layer (surface or bottom), season (hypoxia or normoxia), and their interaction with different modeling for the random effects

	Fixed effects			Random effects
	Estimate	SE	*p*	Variance
*(a)*				
Intercept	**−0.838**	**0.422**	.**047**	2.81
Layer (bottom)	0.228	0.225	.311	0.489
Season (normoxia)	−0.409	0.216	.058	0.462
Layer (bottom): season (normoxia)	**0.643**	**0.228**	.**005**	0.328
*(b)*				
Intercept	−0.857	0.429	.056	3.20
Layer (bottom)	0.245	0.248	.323	0.671
Season (normoxia)	−0.387	0.244	.112	0.676
Layer (bottom): season (normoxia)	**0.642**	**0.179**	**<.001**	–

Note

(a) Species‐specific random effects were modeled for the intercept, and slopes of the three explanatory variables. (b) Species‐specific random effects were modeled for the intercept and slopes of independent effect of layer and season only (not for the interaction). Estimated coefficients, standard errors (SE), *p*‐values of the fixed effects, estimated variance of the random effect are shown. Significant (*p* < .05) estimates are shown with bold. The likelihood ratio test indicates that the fits of these models were not significantly different (χ^2^ = 2.56, df = 1, *p* = .109).

We found a significant interactive effect of water layer and water depth of sampling sites on species composition (PERMANOVA, *F*_1, 225_ = 2.32; *R* ^2^ = 0.010; *p* = .003), as well as their independent effects (layer: *F*_1, 225_ = 9.25; *R* ^2^ = 0.039; *p* < .001; water depth: *F*_1, 225_ = 6.56; *R* ^2^ = 0.027; *p* < .001). Indeed, in the NMDS space, distance between the centroids of surface and bottom layers of samples obtained at deeper sites (upper eight sites among 16 in total) was 0.479, while that of samples of shallower sites (lower eight sites) was 0.401 (Figure S6). This suggests that the compositional difference between bottom and surface layers was smaller at shallower sites.

## 4 DISCUSSION

Species sorting, one of the metacommunity paradigms, predicts that environmental heterogeneity is a major source of compositional variation. Based on pervasive understanding that environmental filtering works more in abiotically severer habitats (Chase, 2007; Daniel et al., 2019; Guo et al., 2014; Lepori & Malmqvist, 2009), among‐habitats compositional variation is expected to be greater when the environmental heterogeneity is so large that the gradient encompasses the most severe habitats (Figure 1a). In the present study system, extremely low DO concentration (<2 mg/L) in the bottom water during summer (Figure S1) served as the severe condition, decreasing the species richness in the bottom layer (Figure S3). As tolerating such extreme oxygen conditions requires certain morphological and physiological traits (Bickler & Buck, 2007; Mandic et al., 2009), different sets of species were likely at surface and bottom layers. However, against this expectation, we found that species composition was more similar during the hypoxic season (Figure 2). The null model analysis indicated that this pattern was robust even after accounting for the effect of sampling bias from the species pool (Figure 3).

### 4.1 Why less compositional variation during hypoxia?

The result that species composition was more similar during hypoxia despite larger environmental heterogeneity (Figure 2) may appear paradoxical, as the hypoxic condition at the bottom water is expected to sort species with adapted traits and filter out the other species. Nevertheless, a series of evidence can explain this counterintuitive result. For example, Pihl et al. (1991) showed that three demersal fish species differed in their response to bottom hypoxia in summer in Chesapeake Bay, USA. However, when the oxygen level fell below the threshold (2 mg/L), all three species similarly showed shifts in their distribution from deep to shallow waters (Pihl et al., 1991). Likewise, Eby and Crowder (2002) found that while the threshold for avoiding hypoxia varied among 10 fish species, it was larger than 2 mg/L for all species. Therefore, it is likely that, although the decrease in DO in the bottom does serve as a filter for species composition, once it exceeds a certain level of severity, fish behaviors (e.g., survival, foraging and growth) will be adversely affected regardless of species identity, at which point limited oxygen no longer plays a role in species sorting. Indeed, in our results, most of the common fish species (14 out of 17) showed a shift of their occurrence frequency from the bottom to surface layer (Figure 4). Moreover, our GLMM indicated that the random variation of the coefficient of the interaction term between water layer and season was smaller than that of their independent effects (Table 1a), and the species‐specific coefficients were in the same direction for all the common species (Figure S4). In addition, the two GLMMs, with and without the random variation in the coefficient of the interaction term, yielded similar fittings. These results collectively imply that even though species differ in their occurrence probability at different layers and seasons (as shown by the estimated large variance of random effects, Table 1), responses to the bottom hypoxia (i.e., less frequent occurrence in the bottom layer in hypoxia) do not vary much across species. Earlier experimental and observational studies have suggested that, for some of the common species in our dataset, behavioral, physiological, or distributional responses to hypoxia were seen at the DO value similar to our threshold (2 mg/L): *Engraulis japonicus* (1.12–2.36 mg/L, Oda et al., 2018), *Mugil cephalus* (2 mg/L, Wannamaker & Rice, 2000), *Sardinops sagax* (2.0–3.0 mg/L, Kreiner et al., 2009), *Scomberomorus niphonius* (4.0 mg/L, Shoji et al., 2005), *Trachurus japonicus* (2 mg/L, Yamamoto, 1991), while information was not available for the other species. Partially supporting our conclusion that the effect of bottom hypoxia is nonspecies specific, a meta‐analysis revealed that effects of hypoxia on 29 fish species were not well explained by species ecological characteristics (Hrycik et al., 2017). Our results and other literature collectively support our second hypothesis that species sorting is weak during hypoxia because extremely low DO concentration harms fish irrespective of the species identity.

The observed shifts in the relative occurrence frequency from the bottom to surface waters during hypoxia can be a result of upward movements of individuals or higher mortality in the bottom (Diaz & Rosenberg, 2008; Levin, 2003). As fish are highly mobile, they can respond sharply to the environmental changes by avoiding unfavorable conditions. Therefore, when the bottom water was hypoxic in summer, they could move upward to escape from the stressful habitat (Eby & Crowder, 2002; Pihl et al., 1991). This would be particularly true for small pelagic fish such as those frequently observed in this study. Moreover, we observed increases in species richness per sample in the surface water during hypoxia, as well as the decreases in the bottom (Figure S3), suggesting that species at the bottom layer moved up to the surface during hypoxia. Meanwhile, evidence seems relatively weak for the case that higher mortality in the bottom is responsible for the observed shifts in the species occurrences. If so, species richness should have only decreased in the bottom and not increased in the surface, contrary to our result (Figure S3). Nevertheless, some species, especially demersal fish, may have experienced higher mortality rate. For example, *Branchiostegus japonicus* whose habitat is specialized to the bottom (M. Ishii & H. Mita, personal communication) almost disappeared during the hypoxia period (Figure S4) and its occurrence did not recover after the hypoxic condition improved, suggesting its higher mortality during hypoxia. Although being beyond the scope of this paper, comparing the responses to bottom hypoxia between demersal and small pelagic fish species would be a fruitful direction of future studies.

### 4.2 Implications for community assembly studies

Our results have general implication in the context of community assembly. We found that fish compositional difference between surface and bottom layers in a bay was smaller during hypoxia season when the environmental heterogeneity was large. This was likely because the extremely hypoxic condition at the bottom induced mass‐effect like distributional shifts from the surface and surface layers irrespective of the species identity, or fish are similarly adversely affected by the severity and thus species differences are small (i.e., more neutral) at the bottom. Although the result appears as conflicts with the prevailing view that environmental heterogeneity leads to larger compositional variation and species sorting plays a more important role in stressful habitats (Chase, 2007; Daniel et al., 2019; Guo et al., 2014; Lepori & Malmqvist, 2009), a limited number of studies agrees with our finding. Lepori and Malmqvist (2009) have found that the deterministic control in macroinvertebrate communities in streams is strongest at the intermediate level of disturbance. The authors concluded that while frequent disturbance strengthened species sorting by selecting disturbance‐tolerant species, extremely intense disturbance might induce extinction of individuals irrespective of species traits, which would hamper the species sorting. Similarly, Kim (2019) pointed to a possibility that species living in extremely severe environments share similar tolerance traits. Therefore, environmental filtering may no longer play a role in such “neutral” communities (Kim, 2019). Based on our finding and previous studies, we speculate that while the increasing environmental severity surely strengthens the filtering on species composition by sorting out vulnerable species, once it exceeds a certain level, the filtering would no longer work because the extreme severity of an environment affects all species in a similar way. Importantly, this may be especially the case when organisms possess high mobile ability which allows them to respond to the environmental stress and move to preferable habitats easily. Future studies should explore the community assembly patterns along environmental gradients, hopefully in systems characterized by higher dispersal rates, to obtain general understanding of community assembly in severe and heterogeneous environments.

### 4.3 Caveats and utility of eDNA metabarcoding analyses

Although the ability of eDNA metabarcoding analyses to detect species from environmental samples has been established (Sigsgaard et al., 2017; Thomsen et al., 2012; Yamamoto et al., 2017), this technique has rarely been applied to the evaluation of community assembly processes. In this study, the eDNA metabarcoding successfully reveals how the compositional variation changes in response to seasonal environmental shift, which illustrates its potential to be applied to community assembly studies. However, there remain several challenges for the technique to be widely applied. First, how accurately the eDNA signal reflects the actual species distribution is still an important issue to be explored. While its relatively short persistence and low diffusion rate promise its utility for detecting “snapshot” distribution (Murakami et al., 2019; Port et al., 2016; Thomsen et al., 2012; Yamamoto et al., 2016), whether the sampling is designed to meet the resolution must be examined carefully. For example, although our monthly sampling at sufficiently distant locations (>4 km apart) apparently enabled us to distinguish the temporal and spatial (horizontal) compositional differences, some of our sampling sites were very shallow, where the vertical mixing of eDNA potentially biases the detected distribution of species. Indeed, we found that the compositional difference between surface and bottom layers detected using eDNA metabarcoding analysis was smaller at shallower sites (Figure S4), suggesting the existence of such a bias at shallow locations. Nevertheless, our main finding that compositional difference between surface and bottom layers was smaller during hypoxia is opposed to the pattern expected from the vertical mixing of eDNA. Since water exchange between bottom and surface is reduced as water columns form, the vertical mixing of eDNA and consequently mixing of species composition are unlikely during hypoxia. Therefore, we conclude that biases due to vertical mixing of eDNA have, if any, a limited influence on our finding. Second, the dataset we used contained only presence–absence information, which would have missed some important information. Estimating species abundance data from eDNA samples is challenging at the present as the correlation between eDNA concentration and abundance of target fish species has not been established under natural conditions (Yates et al., 2019). To avoid potential biases, we did not use the read number of DNA as an abundance index. Third, we note that we conducted only one replication for field sampling and PCR, while it has been generally recommended to obtain multiple replicates for the robust estimates of biodiversity and species composition (Stauffer et al., 2021; Zinger et al., 2019). As the logistical limitations prevented us to design such sound sampling and experimental designs, there potentially exist errors in our dataset and the finding may be interpreted with caution. Nevertheless, we anticipate that unless the stochastic errors are related with some of the important variables (e.g., season or water layers), our findings are largely insensitive to the sampling biases. Finally, the detection of eDNA is influenced not only by the presence of the species but also by the degradation and accumulation of the eDNA itself (Barnes & Turner, 2016). For example, the degradation of eDNA after released into water is faster at higher temperatures (Jo et al., 2019; Tsuji et al., 2017), and it has been assumed that eDNA accumulates in surface waters (Eichmiller et al., 2014; Moyer et al., 2014). We consider that these biases are not important here or at least do not change our main finding, because species richness in surface water was high in summer when the degradation is expected to be faster (Figure S3), and the richness was generally lower in the surface layer where the eDNA accumulation is expected (Figure S3).

## 5 CONCLUSION

In ecosystems, species assemblages vary according to environmental heterogeneity, but to a variable extent. We found that, in a coastal ecosystem, despite the larger environmental heterogeneity during the hypoxic season, species composition was more similar between bottom and surface layers, likely because the adverse effect of hypoxia was nonspecies specific. This result may seem to be contradictory to the current pervasive view that severe environment acts as strong species sorting. However, we speculate that these two contrasting conclusions are compatible: While the increasing environmental severity surely strengthens the filtering by sorting out vulnerable species, once it exceeds a certain level, the filtering would no longer work because too severe environment harms all the species in a similar way. Therefore, species sorting by environment may be greatest at the intermediate level of environmental severity, a hypothesis to be further confirmed in the future studies. Furthermore, by applying the eDNA metabarcoding analyses for examining community assembly processes for the first time to our best knowledge, this study highlights the potential applications of this promising technique across a wide range of disciplines of ecology.

## CONFLICT OF INTEREST

Authors declare no competing interests regarding this study.

## AUTHOR CONTRIBUTION

**Naoto Shinohara:** Conceptualization (lead); Data curation (lead); Formal analysis (lead); Visualization (lead); Writing – original draft (lead); Writing – review & editing (lead). **Yuki Hongo:** Data curation (supporting); Funding acquisition (equal); Investigation (lead); Writing – review & editing (equal). **Momoko Ichinokawa:** Funding acquisition (equal); Supervision (equal); Writing – review & editing (equal). **Shota Nishijima:** Conceptualization (supporting); Funding acquisition (equal); Supervision (equal); Writing – review & editing (lead). **Shuhei Sawayama:** Conceptualization (supporting); Funding acquisition (equal); Investigation (equal); Writing – review & editing (equal). **Hiroaki Kurogi:** Funding acquisition (equal); Investigation (equal); Project administration (lead); Writing – review & editing (equal). **Yasuyuki Uto:** Funding acquisition (equal); Investigation (equal); Writing – review & editing (equal). **Hisanori Mita:** Funding acquisition (equal); Investigation (equal); Writing – review & editing (equal). **Mitsuhiro Ishii:** Funding acquisition (equal); Investigation (equal); Writing – review & editing (equal). **Akane Kusano:** Funding acquisition (equal); Investigation (equal); Writing – review & editing (equal). **Seiji Akimoto:** Funding acquisition (equal); Methodology (equal); Writing – review & editing (equal).

## Supporting information

Supinfo S1

## Data Availability

The data used in this study are available in the Figshare repository (https://doi.org/10.6084/m9.figshare.19609347).
